# Sporadic Oropouche Infection, Acre, Brazil

**DOI:** 10.3201/eid1502.080401

**Published:** 2009-02

**Authors:** Ana Carolina Bernardes Terzian, Roberta Vieira de Moraes Bronzoni, Betânia Paiva Drumond, Mônica Da Silva-Nunes, Natal Santos da Silva, Marcelo Urbano Ferreira, Márcia Aparecida Sperança, Maurício Lacerda Nogueira

**Affiliations:** Faculdade de Medicina de São José do Rio Preto, São Paulo, Brazil (A.C. Bernardes Terzian, R.V. de Moraes Bronzoni, M.L. Nogueira); IBILCE–São José do Rio Preto, São Paulo (A.C. Bernardes Terzian); Universidade Estadual de Montes Claros, Montes Claros, Brazil (B.P. Drumond); Instituto de Ciências Biológicas/Universidade de São Paulo, São Paulo (M. Da Silva-Nunes, N. Santos da Silva, M.U. Ferreira); Faculdade de Medicina de Marília, São Paulo (M.A. Sperança)

**Keywords:** Oropuche virus, arboviruses, Brazil, letter

**To the Editor:** *Oropouche virus* (OROV), a member of the *Bunyaviridae* family, *Orthobunyavirus* genus, Simbu serogroup, is transmitted to humans in urban areas by the biting midge *Culicoides paraensis* and causes epidemic acute febrile disease (*1*). Since its first isolation in Trinidad in 1955 (*2*), OROV has been associated with large outbreaks in South and Central America; half a million cases have been described during the past 45 years (*1*). The tripartite genome of OROV comprises single-strand, negative-sense large (L), medium (M), and small (S) RNAs that encode RNA polymerase, glycoproteins, and nucleocapsid, respectively. Studies have indicated the existence of 3 genotypes of OROV circulating in Brazil: genotypes I and II in the Amazon Basin and genotype III in the Southeast Region (*3*–*5*).

OROV causes explosive urban epidemics. Serologic evidence of exposure to OROV in populations not affected by known outbreaks suggests that the virus circulates endemically (*1*). However, no sporadic infections have been reported. Here we report a sporadic OROV infection detected by clinical and laboratory surveillance of acute febrile illnesses in Acre, a state in the western Amazon region of Brazil. From March 2004 through October 2006, we prospectively investigated 69 febrile episodes in persons 6–60 years of age (mean, 28.1 years) living in the town of Acrelândia (10°13′W, 67°00′S) and surrounding rural areas (25.7% and 74.3% of the sample, respectively).

Serum samples for reverse transcription–PCR (RT-PCR) were stored in liquid nitrogen in the field and shipped on dry ice to the laboratory in São José do Rio Preto, 3,500 km southeast of Acre. Because malaria and several arboviruses are locally endemic (*6*), all patients were screened for malarial parasites by thick-smear microscopy and for flaviviruses and alphaviruses by multiplex-nested RT-PCR (*7*). The samples negative for both malaria and other arboviruses were further tested for OROV with primers targeting the S segment of the OROV genome in a seminested RT-PCR strategy (R.V.M. Bronzoni et al., unpub. data; primers and protocol available from the authors by request). The sample also was isolated in Vero cells, and the RT-PCR described by Moreli et al. (*8*) was used for confirmation.

We sequenced amplicons by using the same primers used for RT-heminested amplification and by using BigDye Terminators version 3.1 (ABI, Foster City, CA, USA) in ABI377 automated sequencer. Sequences were edited by DSGene 2.0 (Accelrys, San Diego, CA, USA) and deposited in GenBank (accession no. EU561644). One (1.4%) of 69 samples tested for OROV by heminested PCR was positive. This sample (BR/2004/ACRE27) was collected from a male patient from a rural area in April 2004. Precautions were followed to avoid contamination; positive and negative controls were used in all reactions; and the procedure was reproduced several times. The patient had ill-defined, mild flu-like symptoms; low-grade fever; and nasal discharge but reported no headache or other major symptoms. He recovered without complication.

We built a phylogenetic tree on the basis of the 522 nucleotide sequences (27–200 aa) of nucleocapsid protein gene of OROV sample BR/2004/ACRE27 and other GenBank sequences from different OROV genotypes. We used sequences from Aino, Akabane, and Tinaroo viruses as the outgroup. A phylogenetic analysis was performed by the neighbor-joining method by using the Kimura 2-parameter nucleotide substitution model (*9*).

The tree showed 3 main clades, corresponding to genotypes I, II, and III, and BR/2004/ACRE27 grouped within genotype I strains (Figure). Both genotypes I and II have been described in OROV outbreaks in Acre; genotype I, however, is found mostly in Pará in the eastern part of the Brazilian Amazon region.

**Figure:**
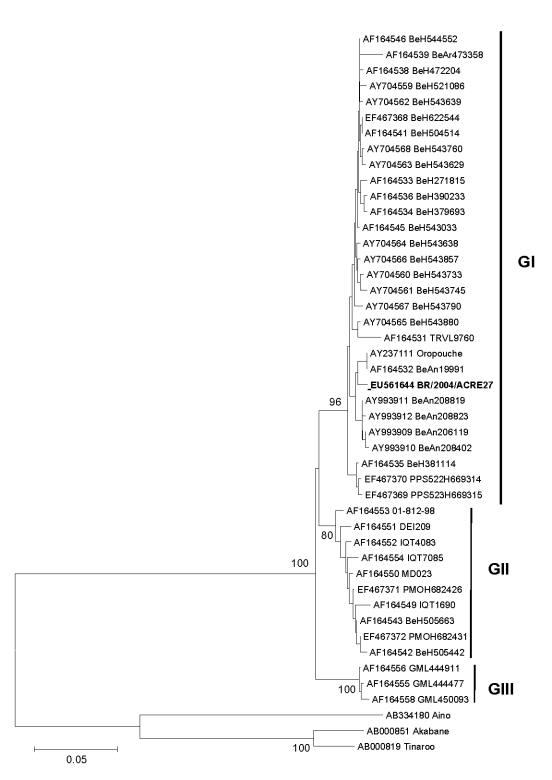
Phylogenetic tree of Oropouche virus strains; **boldface** shows the sample from the patient in this study. Phylogenetic tree was constructed from partial nucleocapsid gene sequence (522 nt, 27–200 aa) by neighbor-joining method implemented in MEGA 3.0 software (*9*). Kimura 2-parameter nucleotide substitution model was used, and the reliability of the branching patterns was tested by 1,000 bootstrap pseudo replicates. Bootstrap values (%) are shown in main nodes. *Aino*, *Akabane*, and *Tinaroo* viruses were used as the out group. The scale bar represents 5% nucleotide sequence divergence. GenBank accession numbers are provided and are grouped by strain designation. GI, genotype I; GII, genotype II; GIII, genotype III.

A baseline serologic survey in rural Acrelândia during March and April 2004 detected antibodies to OROV in 6 (1.7%) of 357 persons 5–90 years of age who were examined by microplaque hemagglutination inhibition (*10*). Because none of these persons had been exposed to known OROV outbreaks in Acre or elsewhere, these findings further suggest the sporadic circulation of OROV in the area.

We describe a sporadic infection of OROV infection in the Amazon region of Brazil in a mildly symptomatic patient. The nucleocapsid gene of the isolate has been sequenced, placing it in the genotype I group, the most commonly found in the Amazon Basin. These data suggest that OROV circulation may be sporadic and clinically silent and, when not associated with outbreaks, most likely neglected by local physicians.

## References

[1] Pinheiro FP, Travassos da Rosa APA, Vasconcelos PFC. Oropouche fever. In: Feigin RD, editor. Textbook of pediatric infectious diseases. Philadelphia: WB Saunders Co.; 2004. p. 2418–23.

[2] Anderson CR, Spence L, Downs WG, Aitken THG. Oropouche virus: a new human disease agent from Trinidad, West Indies. Am J Trop Med Hyg. 1961;10:574–8. 13683183 10.4269/ajtmh.1961.10.574

[3] Saeed MF, Wang H, Nunes M, Vasconcelos PF, Weaver SC, Shope RE, Nucleotide sequences and phylogeny of the nucleocapsid gene of Oropouche virus. J Gen Virol. 2000;81:743–8. 10675412 10.1099/0022-1317-81-3-743

[4] Nunes MR, Martins LC, Rodrigues SG, Chiang JO, Azevedo RSS, da Rosa AP, Oropouche virus isolation, southeast Brazil. Emerg Infect Dis. 2005;11:1610–3. 16318707 10.3201/eid1110.050464 PMC3366749

[5] Azevedo RSS, Nunes MRT, Chiang JO, Bensabath G, Vasconcelos HB, Pinto AYN, Reemergence of Oropouche fever, northern Brazil. Emerg Infect Dis. 2007;13:912–5. 17553235 10.3201/eid1306.061114 PMC2792853

[6] Silva-Nunes M, Malafronte RS, Luz BA, Souza EA, Martins LC, Rodrigues SG, The Acre Project: the epidemiology of malaria and arthropod-borne virus infections in a rural Amazonian population. Cad Saude Publica. 2006;22:1325–34. 10.1590/S0102-311X2006000600021 16751971

[7] de Morais Bronzoni RV, Baleotti FG, Nogueira RMR, Nunes M, Figueiredo LTM. Duplex reverse transcription-PCR followed by nested PCR assays for detection and identification of Brazilian alphaviruses and flaviviruses. J Clin Microbiol. 2005;43:696–702. 10.1128/JCM.43.2.696-702.2005 15695666 PMC548032

[8] Moreli ML, Aquino VH, Cruz AC, Figueiredo LT. Diagnosis of Oropouche virus infection by RT-nested-PCR. J Med Virol. 2002;66:139–42. 10.1002/jmv.2122 11748670

[9] Kumar S, Tamura K, Nei M. MEGA3: Integrated software for molecular evolutionary genetics analysis and sequence alignment. Brief Bioinform. 2004;5:150–63. 10.1093/bib/5.2.150 15260895

[10] Shope RE. The use of a micro-hemagglutination test to follow antibody response after arthropod-borne virus infection in a community of forest animals. [Rio J]. Ann Microbiol. 1963;11:167–71.

